# Gelseminum Sempervirens

**Published:** 1876-08

**Authors:** 


Gelseminum Sempervirens.—The Nos. of the *Lancet* for January 22d and March 18th, contained an account of some experiments made by Dr. Sydney Ringer and Mr. Wm. Murrell on the action of this article. These experiments show:

"1. That, in animals, gelseminum primarily affects respiration. 2. That it reduces the frequency of the respiratory act. 3. That the affection of respiration appears before the paralysis, and passes off earlier. 4. That gelseminum causes death by asphyxia. 5. That, if artificial respiration be maintained until the drug can be eliminated, recovery will take place.

"Such are the facts. What is their explanation? On what part of the respiratory system does the drug exert its influence? Dr. Burdon-Sanderson has kindly consented to make the experiments necessary for the solution of these problems, and will publish the results in the ensuing paper."

